# TMT-Based Quantitative Proteomic Analysis Reveals the Crucial Biological Pathways Involved in Self-Incompatibility Responses in *Camellia oleifera*

**DOI:** 10.3390/ijms21061987

**Published:** 2020-03-14

**Authors:** Yifan He, Qianqian Song, Yuefeng Wu, Shutao Ye, Shipin Chen, Hui Chen

**Affiliations:** 1 Forestry College, Fujian Agriculture and Forestry University, Fuzhou 350002, Fujian, China; yifanhe20181@163.com (Y.H.); qianqiansong2018@163.com (Q.S.); yuefengwu01@163.com (Y.W.); yeshutaosir@163.com (S.Y.); Shipin.fjcsp@126.com (S.C.); 2 Forestry College, Oil Tea Research Center of Fujian Province, Fujian Agriculture and Forestry University, Fuzhou 350002, Fujian, China; 3 Department of Molecular, Cell & Developmental Biology, University of California, Los Angeles, CA 90095, USA

**Keywords:** *Camellia oleifera*, proteomic, pathway, self-incompatibility, TMT

## Abstract

*Camellia oleifera* is a valuable woody oil plant belonging to the Theaceae, *Camellia* oil extracted from the seed is an excellent edible oil source. Self-incompatibility (SI) in *C. oleifera* results in low fruit set, and our knowledge about the mechanism remains limited. In the present study, the Tandem mass tag (TMT) based quantitative proteomics was employed to analyze the dynamic change of proteins response to self- and cross-pollinated in *C. oleifera*. A total of 6,616 quantified proteins were detected, and differentially abundant proteins (DAPs) analysis identified a large number of proteins. Combined analysis of differentially expressed genes (DEGs) and DAPs of self- and cross-pollinated pistils based on transcriptome and proteome data revealed that several candidate genes or proteins involved in SI of *C. oleifera*, including polygalacturonase inhibitor, UDP-glycosyltransferase 92A1-like, beta-D-galactosidase, S-adenosylmethionine synthetase, xyloglucan endotransglucosylase/hydrolase, ABC transporter G family member 36-like, and flavonol synthase. Venn diagram analysis identified 11 proteins that may participate in pollen tube growth in *C. oleifera*. Our data also revealed that the abundance of proteins related to peroxisome was altered in responses to SI in *C. oleifera*. Moreover, the pathway of lipid metabolism-related, flavonoid biosynthesis and splicesome were reduced in self-pollinated pistils by the Kyoto Encyclopedia of Genes and Genomes (KEGG) pathway analysis. In summary, the results of the present study lay the foundation for learning the regulatory mechanism underlying SI responses as well as provides valuable protein resources for the construction of self-compatibility *C. oleifera* through genetic engineering in the future.

## 1. Introduction

Self-incompatibility (SI) exists in most angiosperms as an evolutionary mechanism that stimulates outcrossing and inhibits inbreeding [1]. As a genetically controlled mechanism, SI provides a high level of heterozygosity by overcoming inbreeding depression in flowering plants [2]. Pollen/pollen tube of self-fertilization was recognized and rejected through evolved genetic systems of expressing the same allelic specificity either with style or ovular vicinity and post-fertilization, resulting in seed set inhibited [3]. In addition to the role of SI in intercellular communication research [3,4], it is also an important feature in agronomy [5,6]. SI could occur in all stages from stigma through ovule, therefore it is sure that different plant species might evolve different SI systems [7]. The SI mechanism was revealed in limited planta families because of the extensive properties of SI in the flowering plant [8]. According to genetic characteristics, Self-incompatibility models were commonly classified into gametophytic SI (GSI) and sporophytic SI (SSI) [9]. Mainly well-defined SI models were determined on S-locus. There are no less than two genes on the S-loci, one of them is the male determinate factor, and the other one is the female determinate factor [10]. SSI of *Brassicaceae* is controlled by the stigma receptor kinase [11,12] via interaction with the pollen coat cysteine-rich protein [13,14] and is then phosphorylated intracellularly [15,16]. In *Rosaceae*, *Solanaceae*, and *Plantaginaceae*, GSI was accomplished by male determinate factor S-locus F-box protein/S haplotype-specific F-box protein gene (SLF/SFB) and female determinate factor S-RNase [17,18,19]. Other genes except S-locus were still believed to play important roles in SI [20]. In the *Papaveraceae*, programmed cell death (PCD) contributed to GSI through phosphorylation, ubiquitination, and calcium-dependent signaling, resulting in the termination of pollen tube growth [21]. Late self-incompatibility (LSI) is defined based on the ovary of which inhibition happened, thus different from the other two SI models [22]. The mechanism of LSI is still unclear although LSI is more widespread in the flowering plant because of its evolution position [8,9]. LSI could occur at both the pre- and post-fertilization phases depending on different plant species [23]. Bottleblock of revealing the molecular mechanism exists on these long life span LSI plants because of the difficulty in conducting the molecular genetic test [8,24]. Nevertheless, several kinds of research confirmed the genetic basis of LSI is hypothesized to be gametophytic [23,25,26]. It has been demonstrated that the LSI mechanism in some plants was polygenic and polymorphic according to crossing experiments [9,25,27]. Unfortunately, no LSI determinate genes were found to date which prevents our understanding of LSI.

*Camellia oleifera* Abel. is a valuable oil tree species native to China, famous for its extensive utilization and long history of cultivation [28]. *C. oleifera* is grown principally for its seed and is utilized to provide the *Camellia* oil. The best-treasured product of *C. oleifera* is *Camellia* oil, which is known as ‘‘eastern olive oil’’ as a result of its high quality [29]. SI in *C. oleifera* usually results in a low rate of seed set, which limits the yield. Hence, it is necessary to reveal the molecular mechanism of SI in *C. oleifera* to improve strategies for breeding to guide the improvement of breeding strategies to enhance the yield in the future. In *C. oleifera*, recent evidence suggests that self-fertilized pollen can form pollen tubes and growth down through the style, but the self-fertilized pollen fails to complete double fertilization in the ovary [6,30]. YIFAN et al. compared the transcriptome of self- and cross-pollinated pistil and found several candidate genes that may participate in *C. oleifera* SI [30]. However, knowledge about the molecular mechanism of SI in *C. oleifera* is still limited to date. In the present study, tandem mass tag (TMT) based quantitative proteomics was employed to analyze the dynamic change of proteins response to self- and cross-pollination in *C. oleifera*, which could provide us more information on the protein level about SI response. 

## 2. Results

### 2.1. Identified Protein Overview

**Figure 1:**
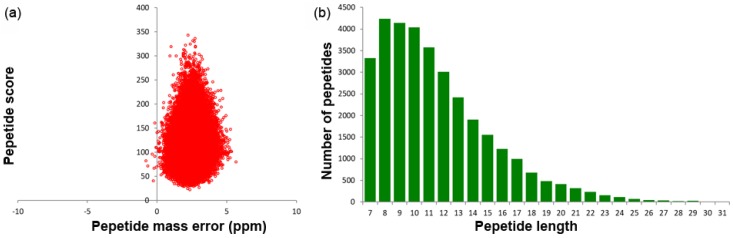
MS identified information based on proteomics analysis: (**a**) Average peptide mass error; (**b**) All identified peptides length distribution.

TMT-based quantitative proteomics was performed to obtain the dynamic change of proteins between self- and cross-pollination pistils in *C. oleifera*. After quality inspection, obtaining a total of 318,969 (54,545 matched) spectra, of which 33,088 were identified peptides (31,071 unique peptides) and 7666 were identified proteins (6616 quantified proteins) (Table 1). The average peptides mass error was less than 10 ppm, suggesting MS data has very high mass accuracy (Figure 1a). The majority of the peptides identified were 7 to 21 amino acid residues in length (Figure 1b), which indicated that those samples satisfied the requiring criteria. Supplementary Table S1 showed the identified protein detail information.

### 2.2. Protein Annotation

Identified protein annotation was performed based on several public databases, containing Gene Ontology (GO) terms, Kyoto Encyclopedia of Genes and Genomes (KEGG) pathways, Protein domain, and Subcellular localization. Detail of information on the identified protein is presented in Supplementary Table S2. 

### 2.3. Identification and Analysis of Differentially Abundant Proteins

**Figure 2:**
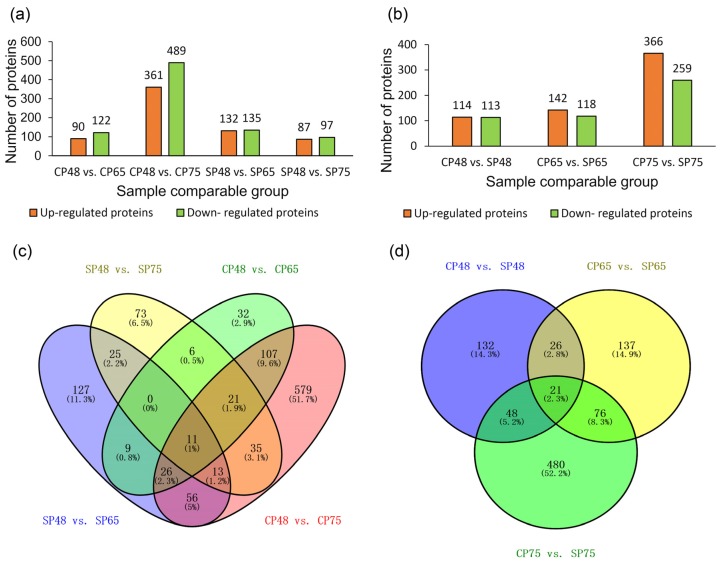
Differentially abundant proteins (DAPs) in all compared groups: (**a**) The information about DAPs was present in the compared groups of CP48 vs. CP65, CP48 vs. CP75, SP48 vs. SP65, and SP48 vs. CP75. (**b**) The information about DAPs was present in the compared groups of SP48 vs. CP48, SP65 vs. CP65, and SP75 vs. CP75. (**c**) DAPs were analyzed by the Venn diagram in the compared groups of CP48 vs. CP65, CP48 vs. CP75, SP48 vs. SP65, and SP48 vs. CP75. (**d**) DAPs were analyzed by the Venn diagram in the compared groups of SP48 vs. CP48, SP65 vs. CP65, and SP75 vs. CP75. CP48 represents 48 h after cross-pollination, CP65 represents 65 h after cross-pollination, CP75 represents 75 h after cross-pollination, SP48 represents 48 h after self-pollination, SP65 represents 65 h after self-pollination, and SP75 represents 75 h after self-pollination.

Differentially abundant proteins (DAPs) were those meeting the criteria (fold change ≥ 1.2 and *P* < 0.05) under comparison of CP48 vs. CP65, CP48 vs. CP75, SP48 vs. SP65, SP48 vs. CP75, CP48 vs. SP48, CP65 vs. SP65, and CP75 vs. SP75. Within cross-pollination, 212 DAPs were identified in CP48 vs. CP65, 122 of which were up-regulated and 90 of which were down-regulated; under CP48 vs. CP75, 850 DAPs were identified, 489 of them were up-regulated and 361 of them were down-regulated (Figure 2a). Within self-pollination, 267 DAPs were identified in SP48 vs. SP65, 135 of which were up-regulated and 132 of which were down-regulated; under SP48 vs. SP75, among the 184 DAPs identified, 97 of them were up-regulated and 87 of them were down-regulated (Figure 2a). In total, 227, 260, and 625 DAPs were identified in CP48 vs. SP48, CP65 vs. SP65, and CP75 vs. SP75, respectively. Under CP48 vs. SP48, 227 DAPs were identified, 114 of them were up-regulated and 113 of them were down-regulated. Among the 260 identified DAPs under CP65 vs. SP65, 142 proteins were up-regulated and 118 proteins were down-regulated. Of 625 DAPs identified in SP75 compared with CP75, 366 proteins were up-regulated and 259 proteins were down-regulated (Figure 2b). 

Venn diagram analysis was created under CP48 vs. CP65, CP48 vs. CP75, SP48 vs. SP65, and SP48 vs. CP75 (Figure 2c). Eleven proteins were commonly differentially abundant in all these compared groups, which may primarily participate in pollen tube growth in *C. oleifera*. In addition, to identify the common and specifically differentially abundant proteins participating in the SI mechanism, a Venn diagram was generated within CP48 vs. SP48, CP65 vs. SP65, and CP75 vs. SP75 (Figure 2d). It is shown that 132, 137, and 480 proteins were differentially abundant in CP48 vs. SP48, CP65 vs. SP65, and CP75 vs. SP75, respectively. A total of 21 common differentially abundant proteins were involved in all three compared groups. 

### 2.4. GO Enrichment Analysis of Pistils between Self- and Cross-Pollinated in C. oleifera

GO enrichment analysis showed that the DAPs including up-regulated proteins and down-regulated proteins between CP48 and CP65 were classified into 46 functional terms (Supplementary Figure S3); DAPs including up-regulated proteins and down-regulated proteins of CP48 vs. CP75 were classified into 52 GO terms (Supplementary Figure S4), while DAPs including up-regulated proteins and down-regulated proteins of SP48 vs. SP65 were classified into 40 GO terms (Supplementary Figure S5); 33 GO terms were obtained based on the DAPs including up-regulated proteins and down-regulated proteins of SP48 vs. SP75 (Supplementary Figure S6). For cellular components, intracellular non-membrane-bounded organelle, non-membrane-bounded organelle, ribonucleoprotein complex, intracellular ribonucleoprotein complex, and ribosome were commonly found in all four compared groups; for molecular function, structural molecule activity was commonly found in all four compared groups; for biological processes, oxidation-reduction process was commonly found in all four compared groups (Table 2). The results indicated that those common GO terms might be related to the pollen tube growth in the pistil of *C. oleifera*.

GO enrichment analysis also revealed that the DAPs including up-regulated proteins and down-regulated proteins between CP48 and SP48 were classified into 34 GO terms (Supplementary Figure S7), while DAPs including up-regulated proteins and down-regulated proteins of CP65 vs. SP65 were classified into 39 GO terms (Supplementary Figure S8); 52 GO terms were created based on the DAPs including up-regulated proteins and down-regulated proteins of CP75 vs. SP75 (Supplementary Figure S9). For cellular components, the DAPs were predominantly distributed in the non-membrane-bounded organelle, intracellular organelle, and intracellular ribonucleoprotein complex between CP48 and SP48; the DAPs of CP65 vs. SP65 were principally enriched in the extracellular region; the DAPs were mainly distributed in chromatin, chromosomal part, and intracellular non-membrane-bounded organelle under CP75 vs. SP75. For molecular function, the DAPs primarily found in structural molecule activity, acyl-CoA dehydrogenase activity, and oxidoreductase activity, acting on the CH-CH group of donors under CP48 vs. SP48; the DAPs largely found in methionine adenosyltransferase activity, lyase activity, and Phosphoenolpyruvate carboxykinase (ATP) activity under CP65 vs. SP65; the DAPs of CP75 vs. SP75 essentially found in enzyme inhibitor activity, catalytic activity, and ATP activity. For biological processes, the DAPs of CP48 vs. SP48 generally participated in the oxidation-reduction process, lipid transport, and cell wall macromolecule catabolic process. The DAPs of CP65 vs. SP65 mainly participated in lipid transport and metal ion homeostasis; the DAPs mostly participated in chromatin assembly or disassembly, single-organism metabolic process, and single-organism carbohydrate metabolic process under CP75 vs. SP75.

### 2.5. KEGG Pathway Enrichment Analysis of Self- and Cross Pollinated Pistils in C. Oleifera

KEGG enrichment analysis showed that the DAPs between CP48 and CP65 were largely enriched in the pathway of ribosome (ath03010), biosynthesis of secondary metabolites (ath01110) and peroxisome (ath04146) (Supplementary Figure S10); DAPs between CP48 and CP75 were primarily enriched in the pathway of ribosome (ath03010), spliceosome (ath03040), and phenylalanine metabolism (ath00360) (Supplementary Figure S11); DAPs between SP48 and SP65 were predominantly enriched in the pathway of flavonoid biosynthesis (ath00941), phenylpropanoid biosynthesis (ath00940), and stilbenoid, diarylheptanoid, and gingerol biosynthesis (ath00945) (Supplementary Figure S12); DAPs between SP48 and SP75 were essentially enriched in the pathway of flavonoid biosynthesis (ath00941), phenylpropanoid biosynthesis (ath00940), and biosynthesis of secondary metabolites (ath01110) (Supplementary Figure S13).

**Figure 3:**
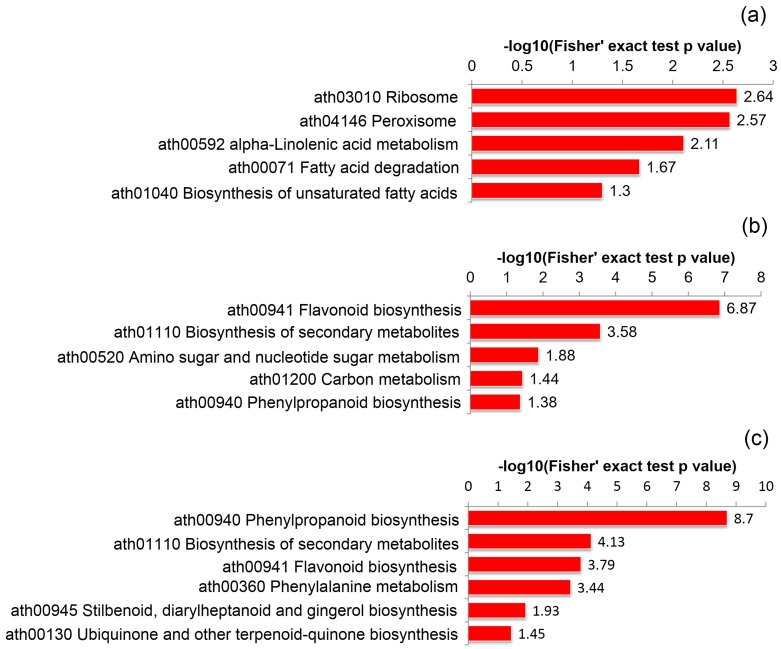
KEGG pathway enrichment analysis of pistils between self- and cross-pollinated in *C. oleifera*: (**a**) KEGG pathway enrichment analysis of pistils at 48 h after self- and cross-pollinated in *C. oleifera*; (**b**) KEGG pathway enrichment analysis of pistils at 65 h after self- and cross-pollinated in *C. oleifera*; (**c**) KEGG pathway enrichment analysis of pistils at 75 h after self- and cross-pollinated in *C. oleifera*.

KEGG enrichment analysis also showed that the DAPs between CP48 and SP48 were mostly enriched in the pathway of ribosome (ath03010), peroxisome (ath04146), and alpha-linolenic acid metabolism (ath00592) (Figure 3a); DAPs between CP65 and SP65 were largely enriched in the pathway of flavonoid biosynthesis (ath00941), biosynthesis of secondary metabolites (ath01110), and amino sugar and nucleotide sugar metabolism (ath00520) (Figure 3b); DAPs between CP75 and SP75 were primarily enriched in the pathway of phenylpropanoid biosynthesis (ath00940), biosynthesis of secondary metabolites (ath01110), and flavonoid biosynthesis (ath00941) (Figure 3c). 

### 2.6. Protein Domain Enrichment Analysis of Pistils between Self- and Cross-Pollinated in C. oleifera

Protein domain enrichment analysis showed that the DAPs between CP48 and CP65 were essentially enriched in AMP-dependent synthetase/ligase, Acyl-CoA dehydrogenase/oxidase C-terminal, and AMP-binding enzyme C-terminal domain (Supplementary Figure S14); DAPs between CP48 and CP75 were largely enriched in L-Aspartase-like, RNA helicase, DEAD-box type, Q motif, and Peptidase C1A, papain C-terminal (Supplementary Figure S15); DAPs between SP48 and SP65 were mostly enriched in L-Aspartase-like, Fumarase/histidase, N-terminal, and Alpha crystallin/Hsp20 domain (Supplementary Figure S16); DAPs between SP48 and SP75 were predominantly enriched in SGNH hydrolase-type esterase domain, GDSL lipase/esterase, and Isopenicillin N synthase-like (Supplementary Figure S17).

Protein domain enrichment analysis also showed that the DAPs between CP48 and SP48 were enriched in Bifunctional inhibitor/plant lipid transfer protein/seed storage helical domain, AMP-dependent synthetase/ligase, and Acyl-CoA oxidase/dehydrogenase, central domain (Supplementary Figure S18); DAPs between CP65 and SP65 were enriched in Alpha crystallin/Hsp20 domain, Isopenicillin N synthase-like, and Oxoglutarate/iron-dependent dioxygenase (Supplementary Figure S19); DAPs between CP75 and SP75 were enriched in L-Aspartase-like, Linker histone H1/H5, domain H15, and Pectinesterase inhibitor domain (Supplementary Figure S20).

### 2.7. Venn Analysis of DEGs and DAPs of Self- and Cross Pollinated Pistils in C. oleifera Based on Transcriptome and Proteome Data

**Figure 4:**
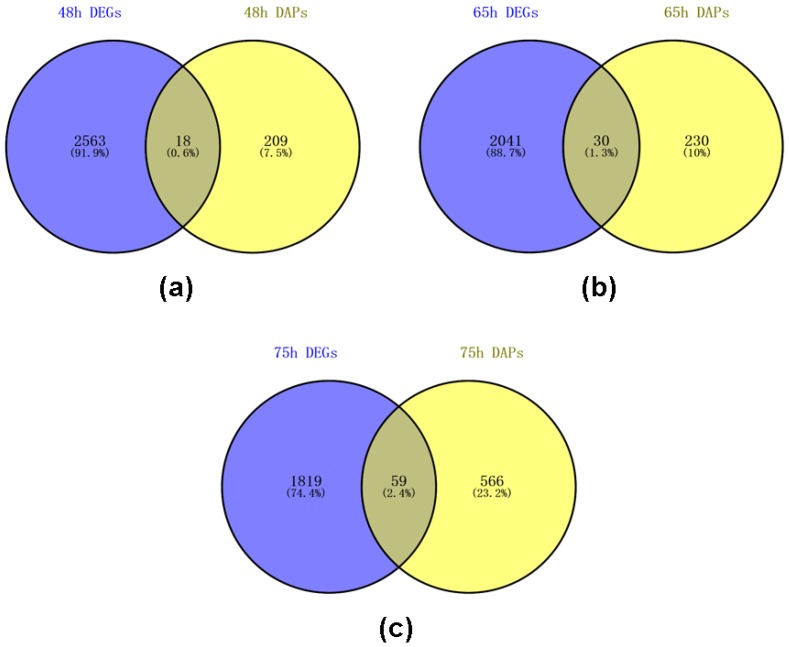
Venn analysis of differentially expressed genes (DEGs) and DAPs of pistils between self- and cross-pollinated in *C. oleifera*: (**a**) Venn analysis of DEGs and DAPs of pistils at 48 h after self- and cross-pollinated in *C. oleifera*; (**b**) Venn analysis of DEGs and DAPs of pistils at 65 h after self- and cross-pollinated in *C. oleifera*; (**c**) Venn analysis of DEGs and DAPs of pistils at 75 h after self- and cross-pollinated in *C. oleifera*.

To better understand the mechanism of SI in *C. oleifera*, Venn analysis was completed based on the previous transcriptome data [30], which have equivalent comparisons of self- and cross-pollination samples collected at the same time. After 48 h of self- and cross-pollinated, 18 DEGs or DAPs were found differentially expressed or differentially abundant in pistils (Figure 4a); 30 DEGs or DAPs were found differentially expressed or differentially abundant in pistils after 65 h of self- and cross-pollinated (Figure 4b); 59 DEGs or DAPs were found differentially expressed or differentially abundant in pistils after 75 h of self- and cross-pollinated (Figure 4c). These DEGs or DAPs could act important role in the SI of *C. oleifera* (Supplementary Table S21), including but not limited to polygalacturonase inhibitor (c122207_g1), UDP-glycosyltransferase 92A1-like (c120823_g1), beta-D-galactosidase (c130743_g2), S-adenosylmethionine synthetase (c131722_g2), xyloglucan endotransglucosylase/hydrolase (c130048_g1), ABC transporter G family member 36-like (c133919_g3), and flavonol synthase (c118197_g1). Gene expression is a multistep process, and any step of gene expression may be modulated, from the DNA-RNA transcription step to the post-translational modification of a protein. Our data revealed a low correlation between transcriptome and proteome as well as a much lower dynamic within the proteome compared to the transcriptome. We cannot exclude that this lower dynamic is at least partly due to the different methods used for the respective transcriptomic and proteomic samples. For example, detection efficiencies and sensitivity limits for each method are not necessarily identical. Some differences between the transcriptome and proteome might be due to an extremely low abundance of transcripts or proteins. Moreover, we believe that translation regulation and post-transcriptional regulation likely plays a major role in determining pistil protein accumulation under cross-pollination and self-pollination in *C. oleifera*. It will be interesting to further explore the regulation of gene expression in self-incompatibility in *C. oleifera*.

## 3. Discussion

### 3.1. Proteins Participated in Pollen Tube Growth in C. oleifera

In the present study, eleven proteins were identified and may involve in pollen tube growth in *C. oleifera* (Table 3). Among them, the protein of c130529_g2 (Ubiquitin-40S ribosomal protein S27a isoform (1) and c82718_g1 (22.7 kDa class IV heat shock protein-like) were down-regulated from 48 h to 65 h after pollinated both in self- and cross-pollinated pistils; the other nine proteins were almost up-regulated from 48 h to 65 h after pollinated both in self- and cross-pollinated pistils. Sigma factor sigb regulation protein rsbq (c102785_g1) is a hydrolase involved in stress regulation [31]. 9-cis-epoxycarotenoid dioxygenase (NCED) is a vital rate-limiting enzyme in the pathway of abscisic acid (ABA) biosynthetic. Previous studies suggested that ABA biosynthesis could be improved by increased transcript levels of NCED in plants [32,33]. It is interesting to note that the increased protein abundance of c121964_g2 (9-cis-epoxycarotenoid dioxygenase 3 protein family isoform (2) may regulate pollen tube growth through regulating ABA accumulation in *C. oleifera* pistil. Peroxidase is a widespread enzyme that oxidizes a large number of reducing compounds. Plant class III peroxidases function in the process of oxidation of toxic reductants, salt tolerance, oxygen stress, auxin catabolism, as well as defense responses [34]. Prior studies that have noted the importance of plant class III peroxidases in SI response of *Senecio squalidus* L.(Asteraceae) [35]. In our work, a class III peroxidase (c124653_g1) was identified and supposed to involve in pollen tube growth in *C. oleifera*. Phenylpropanoids are identified to act as a critical role in pollen fertility [36]. Phenylalanine ammonia-lyase mediated phenylpropanoid metabolism by catalyzing the first step of the phenylpropanoid skeleton biosynthesis [37]. It is reported that receptor-like kinases (RLKs) mediate pollen tube growth and identification [38]. The current study identified a receptor-like protein kinase HAIKU2 (c135487_g1) which encodes leucine-rich repeat (LRR) kinase, but its function in *C. oleifera* is unclear. It would be interesting to further explore these identified proteins function in pollen tube growth in *C. oleifera*.

### 3.2. Peroxisome and Lipid Metabolism Might Act A Key Role in SI Response of C. oleifera

Peroxisomes are multi-functional organelles critical for plant growth and development [39]. Plant peroxisomes involved in numerous physiological processes, containing phytohormone biosynthesis, lipid catabolism, reactive oxygen species metabolism, and many others [40,41,42]. It was previously found that Peroxisome morphology9 (APEM9) mediates pollen maturation/germination of which participating in peroxisome biogenesis and function [43]. Interestingly, our data revealed that several genes involved in the pathway of peroxisome, including catalase-3 (c127728_g1), peroxisomal acyl-coenzyme A oxidase 3 (c119854_g1), hypothetical protein (c121573_g1 and c135528_g1), and peroxisomal acyl-coenzyme A oxidase 1-like (c119759_g2) were down-regulated 48 h after self-pollinated compared with cross-pollinated (Supplementary Table S22). Another interesting finding is that a peroxisome biogenesis protein 19-1-like (c114131_g1) was up-regulated 48 h after self-pollinated compared with cross-pollinated. It was demonstrated that lipids mediate pollen-tube growth by regulating the water flow of pollen [44]. Until recently, there was no genetic evidence to support lipid metabolism is involved in SI response. According to the analysis of the KEGG pathway, the “fatty acid degradation”, “α-Linolenic acid metabolism”, and “biosynthesis of unsaturated fatty acids” were significantly enriched in down-regulated genes 48 h after self-pollination in *C. oleifera* (Supplementary Table S22). In particular, genes participated in fatty acid degradation including a peroxisomal acyl-coenzyme A oxidase 3 (c119854_g1), a glyoxysomal fatty acid beta-oxidation multifunctional protein (c121302_g2), and a peroxisomal acyl-coenzyme A oxidase 1-like (c119759_g2) were down-regulated. Energy for pollen germination and pollen tube elongation usually comes from sugar by fatty acid degradation. In *Arabidopsis* mutants, impairing of fatty acid degradation leads to restriction of pollen tube elongation [45]. It seems that it induces down-regulated fatty acid degradation resulting in energy lacking in SI response in *C. oleifera*. It must also be mentioned that AMP-dependent CoA ligase (c117849_g1), which is involved in α-Linolenic acid metabolism and acyl-coenzyme A thioesterase 8 (c129351_g1), which participated in the unsaturated fatty acids biosynthesis were also down-regulated. As peroxisomes play an essential role in lipid metabolism, combining our research data suggested that these genes involved in peroxisomes and lipid metabolism might cooperate function as candidates for the SI response in *C. oleifera*. 

### 3.3. Flavonoids May Negatively Regulate SI Response in C. oleifera

**Figure 5:**
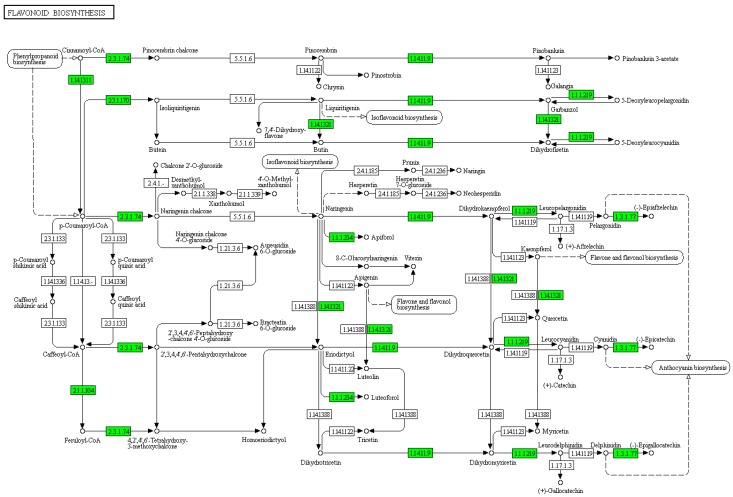
KEGG Pathway analysis of flavonoid biosynthesis 75 h after self- and cross-pollination in *C. oleifera*. The green color indicated decreased protein abundances level in self-pollinated pistils compared with cross-pollinated pistils.

As a class of secondary metabolites, flavonoids can be divided into flavonols, flavones, proanthocyanidins, anthocyanins, and isoflavones [46,47,48]. Flavonoids are essential for plant development and stress tolerance [49,50,51]. The roles of flavonoids in plant reproductive processes were demonstrated [52,53,54,55,56,57,58,59]. Previous studies reported that chalcone synthase-deficient plants present self-incompatibility and male sterility to some extent [60]. As previously described, quercetin and kaempferol produced by flavonoids in tobacco anthers could stimulate pollen tube growth as well as seed development [56]. Previous studies also reported that pollen tubes of flavonol-deficient Petunia show remarkable changes in wall structure resulting in tube disruption [57]. Interestingly, it was found that the abundances of proteins involved in the flavonoid biosynthesis were reduced 75 h after self-pollinated compared with cross-pollinated (Figure 5). It is important to point out that a total of 8 DAPs involved in flavonoid biosynthesis were decreased 75 h after pollination, including chalcone synthase (CHS), flavanone 3-hydroxylase (F3H), flavanol synthase (FLS), dihydroflavonol4-reductase (DFR), and anthocyanidin reductase (ANR) (Supplementary Table S23). This decrease in flavonoid enzymes suggests that flavonoids should be necessary for SI in *C. oleifera*. These results imply that flavonoids may behave as signal molecules negatively regulating SI response in *C. oleifera*.

### 3.4. Splicesome Increased in SI Response in C. oleifera

**Figure 6:**
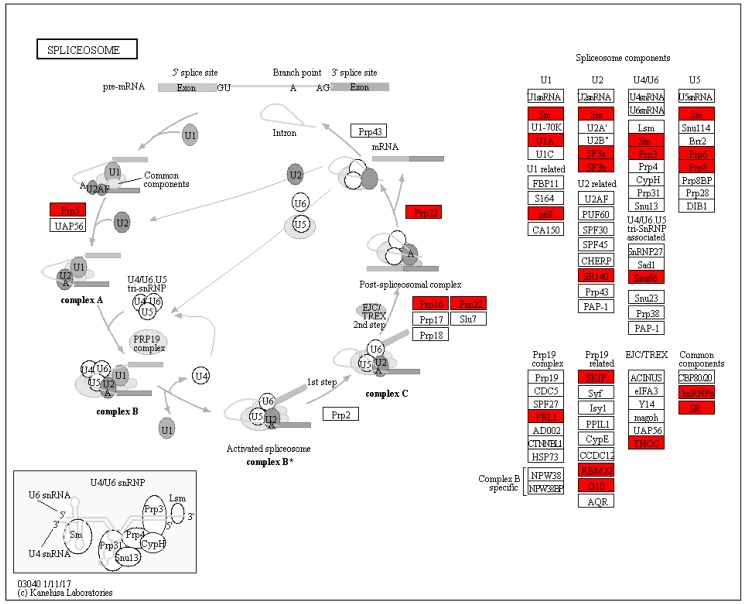
KEGG Pathway analysis of splicesome 75 h after self- and cross-pollination in *C. oleifera*. The red color indicated increased protein abundances level in self-pollinated pistils compared with cross-pollinated pistils.

RNA processing has been demonstrated to play a crucial role in female gametophyte development [61,62]. Recent evidence suggests that Lachesis (LIS) gene influenced synergid cell fate, which is required for splicing through the U4/U6 spliceosome [63]. In addition, Clotho/Gametophytic factor1 (CLO/GFA1) is an important part of the spliceosome of which are involved in suppressing egg cell fate and gametic cell fate [62]. Additionally, Atropos (ATO) encodes the pre-mRNA splicing factor SF3a60 homolog which is involved in female gametophyte function [62]. Slow walker1 (SWA1) encodes a yeast U3 small nucleolar ribonucleoprotein15 (UTP15) homolog that is essential for pre-RNA processing and the mutant of SWA1 leading to complete female sterility in Arabidopsis [64]. One unanticipated finding was that 25 proteins were identified involved in the pathway of splicesome, and all of them were up-regulated 75 h after self-pollinated compared with cross-pollinated (Figure 6 and Supplementary Table S24). These data suggested that RNA processing extensively occurred in response to SI in *C. oleifera*.

### 3.5. Hypothetical Model Occurring in SI Response in C. oleifera

Contrary to expectation, the female determinate factor S-RNase and the male determinate factor S-locus F-box protein of gametophytic self-incompatibility was not involved in SI response in this study. It seems the SI mechanism of *C. oleifera* is different from those that have been revealed in the *Rosaceae*, *Solanaceae*, and *Plantaginaceae* [18,19]. Based on the results presented in this study, we propose a model for the SI responsive pathways in *C. oleifera*. According to this model, self-pollination induced alterations in the pathway of peroxisome, and down-regulated lipid metabolism, which resulted in the energy lacking. In the meantime, flavonoids were synthesis decreased, and RNA processing events increased later after self-pollinated. These changes altogether lead to SI in *C. oleifera*.

## 4. Materials and Methods

### 4.1. Plant Materials

The materials used in the present study include two *C. oleifera* cultivars of “Min 43” and “Min 48”. Then, 25-year-old trees with 2.5 m height were mixed plant in Minhou Tongkou Forest Farm (26°05′ N, 119°17′ E) where the altitude is 186 m, Fujian Province, China.

### 4.2. Pollination Treatment

Pollen of the “Min 43” and “Min 48” cultivars was collected from balloon-stage flowers and stored at 25 °C for eight hours. Self-pollination (SP) of Min 48 × Min 48 and cross-pollination (CP) of Min 48 × Min 43 treatments were performed from 8:00–11:00 am and 1:00–4:00 pm on sunny days in late November. Min 48 was the cultivar donor of pollens and pistils in the self-pollination experiments. In the cross-pollination experiments, Min 43 was the cultivar donor of pollens and Min 48 was the cultivar donor of pistils. Pistils of 48 h, 65 h, and 75 h after SP and CP were harvest and marked as SP48, SP65, SP75, CP48, CP65, and CP75. Samples were collected in liquid nitrogen and stored at −80 °C.

### 4.3. Protein Extraction

We used liquid nitrogen to grind samples into powder and then transfer those powders to a 5 mL centrifuge tube. Next, adding four volumes of lysis buffer (8 M urea, 1% Triton-100, 10 mM dithiothreitol, and 1% Protease Inhibitor Cocktail, 3 μM TSA, 50 mM NAM, and 2 mM EDTA) to the powder, after that sonicated three times on ice by a high-intensity ultrasonic processor (Scientz, Ningbo, Zhejiang, China). The residual debris was discarded through centrifugation at 20,000× *g* for 10 min at 4 °C. Lastly, precipitated the protein in pre-cold 20% TCA at 4 °C for 2 h. The supernatant was discarded after being centrifuged at 12,000× *g* for 3 min at 4 °C. We used cold acetone to wash the residual precipitate three times. Then we redissolved the protein in 8 M urea, and measured the protein concentration by the BCA protein assay kit (Bio-Rad, Hercules, CA, USA). 

### 4.4. Trypsin Digestion

We used 5 mM dithiothreitol to reduce the protein solution at 56 °C for 30 min and 11 mM iodoacetamide to alkylate at room temperature in darkness for 15 min to digestion. After that, we added 100 mM TEAB to a urea concentration of below 2M to dilute the protein sample. Lastly, we added trypsin at a mass ratio of trypsin to the protein of 1:50 to perform the first digestion at 37 °C overnight, and then a second mass of trypsin to protein was 1:100 to perform a second 4 h to digest.

### 4.5. TMT Labeling

After trypsin digestion, the peptide was desalted using the Strata X C18 SPE column (Phenomenex, Torrance, CA, USA) and vacuum-dried. The peptide was reconstituted in 0.5 M TEAB and treated with TMT reagent (Thermo Fisher Scientific, Carlsbad, CA, USA). Concisely, one unit of TMT reagent was thawed and reconstituted in acetonitrile. The peptide mixtures were then incubated at room temperature for 2 h and pooled, desalted, and dried by vacuum centrifugation.

### 4.6. HPLC Fractionation

The trypsin-digested peptides were fractionated by high pH reverse-phase HPLC using Agilent 300Extend C18 column (5 μm particles, 4.6 mm ID, 250 mm length, Agilent, Santa Clara, CA, USA). Firstly, peptides were separated into 60 fractions in a gradient of 8% to 32% acetonitrile (pH 9.0) over 60 min. After that, the peptides were combined into 18 fractions and dried by vacuum centrifuging. 

### 4.7. LC-MS/MS Analysis

The tryptic peptides were dissolved in solvent A (0.1% formic acid in 2% acetonitrile) and applied directly loaded onto a reversed-phase analytical column with 15 cm in length and 75 μm in diameter (Thermo Fisher Scientific, Carlsbad, CA, USA). The gradient including an increase from 6% to 24% solvent B (0.1% formic acid in 90% acetonitrile) over 26 min, 24% to 36% in 8 min, and climbing to 80% in 3 min then holding at 80% for the last 3 min, all performed on an EASY-nLC 1000 UPLC system (Thermo Fisher Scientific, Carlsbad, CA, USA) at a constant flow rate of 350 nL/min. 

The peptides were processed by NSI source and then analyzed by tandem mass spectrometry (MS/MS) in Orbitrap Fusion (Thermo Fisher Scientific, Carlsbad, CA, USA) coupled to UPLC online. The applied electrospray voltage was 2.0 kV. The m/z scan range was 350 to 1550 for a full scan, and intact peptides were detected in the Orbitrap at a resolution of 60,000. Peptides for MS/MS were then selected using the NCE setting as 28 and the fragments were detected in the Orbitrap at a resolution of 15,000. The data acquisition mode uses a dependent scanning (DDA) program, that is, after the first scan, the first 20 peptides with the highest signal intensity are chosen to enter the peptide precursor and enter the HCD collision cell in turn, using 35% of the fragmentation energy. Secondary mass spectrometry was performed in sequence. To improve the effective utilization, set the automatic gain control (AGC) to 5E4, the signal threshold to 5000 ions/s, the maximum injection time to 200 ms, and the dynamic exclusion time of the tandem mass spectrometry scan to 30 seconds to prevent the replication of the parent ion scanning. 

### 4.8. Database Search

We used the Maxquant search engine (v.1.5.2.8) to process the resulting MS/MS data. Tandem mass spectra were searched against the *C. oleifera* transcriptomic database (BioProject ID PRJNA507932) concatenated with the reverse decoy database. Trypsin/P was specified as a cleavage enzyme allowing up to 2 missing cleavages. The minimum length of the peptide was set to 7 amino acid residues; the maximum number of peptide modifications was 5. In the first search, the mass tolerance for precursor ions was set as 20 ppm and 5 ppm in the main search, and the mass tolerance for fragment ions was set as 0.02 Da. Carbamidomethyl on Cys was specified as fixed modification and oxidation on Met was specified as variable modifications. The quantitative method was set to TMT-6plex, and the false discovery rate (FDR) for protein identification and peptide-spectrum match (PSM) identification was set to 1%.

### 4.9. Data Bioinformatics Analyses

#### 4.9.1. GO Annotation

GO annotation proteome was derived from the UniProt-GOA database. First, the identified protein ID converted to UniProt ID, and then mapped to the GO IDs through the protein ID. If some identified proteins were not annotated through the UniProt-GOA database, the InterProScan soft will be used to annotated the GO function of the proteins based on the protein sequence alignment method. After that, proteins were classified based on three categories: biological process, cellular component, and molecular function through Gene Ontology annotation. 

#### 4.9.2. Domain Annotation

We annotated the functional description of the identified protein domains using InterProScan (a sequence analysis application) based on the protein sequence alignment method and used the InterPro domain database. 

#### 4.9.3. KEGG Pathway Annotation

KEGG database was exploited to annotate the protein pathway. First, the KEGG database description of proteins was annotated using the KEGG online service tools KAAS. After that, we used the KEGG online service tools KEGG mapper to map the annotation results to the KEGG pathway database. 

#### 4.9.4. Subcellular Localization

We employed wolfpsort which is a subcellular localization predication soft to predict subcellular localization. An updated version of PSORT/PSORT II used by Wolfpsort to predict eukaryotic sequences. 

#### 4.9.5. Enrichment of Gene Ontology Analysis

Proteins were divided into three categories by GO annotation: biological process, cellular compartment, and molecular function. For each category, a two-tailed fisher’s exact test was utilized to test the enrichment of the differentially accumulated protein against all identified proteins. The GO with a corrected *P*-value < 0.05 is considered significant.

#### 4.9.6. Enrichment of Pathway Analysis

KEGG database was exploited to identify enriched pathways through a two-tailed fisher’s exact test to test the enrichment of the DAPs against all identified proteins. The pathway with a corrected *P*-value < 0.05 was considered significant. 

#### 4.9.7. Enrichment of Protein Domain Analysis

For each category of proteins, the InterPro database was studied and a two-tailed fisher’s exact test was utilized to test the enrichment of the DAPs against all identified proteins. Protein domains with a *P*-value < 0.05 were considered significant. 

## 5. Conclusions

In the present study, TMT-based proteomic analysis was conducted to reveal the protein response to self-pollination and cross-pollination. A total of eleven proteins were identified based on Venn analysis of DAPs and may participate in pollen tube growth in *C. oleifera.* DAPs including polygalacturonase inhibitor, UDP-glycosyltransferase, beta-D-galactosidase, S-adenosylmethionine synthetase, xyloglucan endotransglucosylase/hydrolase, ABC transporter G family member 36-like, and flavonol synthase were remarkably induced responses to SI. Furthermore, KEGG pathway analysis indicated that the pathway of peroxisome, lipid metabolism-related, flavonoids biosynthesis, and splicesome was associated with SI responses in *C. oleifera*.

## Figures and Tables

**Table 1:** Detail information of the identified proteins overview.

Total Spectrums	Matched Spetrums	Peptides	Unique Peptides	Identified Proteins	Quantifiable Proteins
318,969	54,545	33,088	31,071	7666	6616

**Table 2:** Common GO terms under CP48 vs. CP65, CP48 vs. CP75, SP48 vs. SP65, and CP48 vs. CP75.

Cellular Component	
	intracellular non-membrane-bounded organelle
	non-membrane-bounded organelle
	ribonucleoprotein complex
	intracellular ribonucleoprotein complex
	ribosome
Molecular Function	
	structural molecule activity
Biological Process	
	oxidation-reduction process

**Table 3:** Proteins participated in pollen tube growth in *C. oleifera.*

Protein Accession	Protein Description	Subcellular Localization
c102785_g1	Sigma factor sigb regulation protein rsbq	cytoplasm
c121964_g2	9-cis-epoxycarotenoid dioxygenase 3 protein family isoform 2	mitochondria
c124653_g1	Class III peroxidase	chloroplast
c130353_g1	Alpha-copaene synthase	nucleus
c130529_g2	Ubiquitin-40S ribosomal protein S27a isoform 1	nucleus
c131407_g2	Phenylalanine ammonia-lyase	chloroplast
c132935_g1	Formamidase-like isoform 1	nucleus
c134412_g1	Terpene synthase	cytoplasm
c134849_g2	(E)-beta-caryophyllene synthase	cytoskeleton
c135487_g1	Receptor-like protein kinase HAIKU2-like	nucleus
c82718_g1	22.7 kDa class IV heat shock protein-like	chloroplast

## Supplementary Materials

Supplementary Materials can be found at https://www.mdpi.com/1422-0067/21/6/1987/s1. Table S1. The detailed information of identified proteins. Table S2. Protein annotation. Figure S3. GO enrichment analysis of DAPs between CP48 and CP65. Figure S4. GO enrichment analysis of DAPs between CP48 and CP75. Figure S5. GO enrichment analysis of DAPs between SP48 and SP65. Figure S6. GO enrichment analysis of DAPs between SP48 and SP75. Figure S7. GO enrichment analysis of DAPs between CP48 and SP48. Figure S8. GO enrichment analysis of DAPs between CP65 and SP65. Figure S9. GO enrichment analysis of DAPs between CP75 and SP75. Figure S10. KEGG enrichment analysis of DAPs between CP48 and CP65. Figure S11. KEGG enrichment analysis of DAPs between CP48 and CP75. Figure S12. KEGG enrichment analysis of DAPs between SP48 and SP65. Figure S13. KEGG enrichment analysis of DAPs between SP48 and SP75. Figure S14. Protein domain enrichment analysis of DAPs between CP48 and CP65. Figure S15. Protein domain enrichment analysis of DAPs between CP48 and CP75. Figure S16. Protein domain enrichment analysis of DAPs between SP48 and SP65. Figure S17. Protein domain enrichment analysis of DAPs between SP48 and SP75. Figure S18. Protein domain enrichment analysis of DAPs between CP48 and SP48. Figure S19. Protein domain enrichment analysis of DAPs between CP65 and SP65. Figure S20. Protein domain enrichment analysis of DAPs between CP75 and SP75.

## Abbreviations

ABA	Abscisic acid
AGC	Automatic gain control
ANR	Anthocyanidin reductase
APEM9	Peroxisome morphology9
ATO	Atropos
ATP	Phosphoenolpyruvate carboxykinase
CHS	Chalcone synthase
CP48	48 h after cross-pollination
CP65	65 h after cross-pollination
CP75	75 h after cross-pollination
DAPs	Differentially abundant proteins
DEGs	Differentially expressed genes
DFR	Dihydroflavonol4-reductase
FDR	false discovery rate
FLS	Flavanol synthase
GO	Gene Ontology
GSI	Gametophytic SI
KEGG	Kyoto Encyclopedia of Genes and Genomes
LSI	Late self-incompatibility
LRR	Leucine-rich repeat
NCED	9-cis-epoxycarotenoid dioxygenase
PCD	Programmed cell death
PSM	peptide-spectrum match
RLKs	Receptor-like kinase
SFB	S haplotype-specific F-box protein gene
SI	Self-incompatibility
SLF	S-locus F-box protein
SSI	Sporophytic SI
SWA1	Slow walker1
SP48	48 h after self-pollination
SP65	65 h after self-pollination
SP75	75 h after self-pollination
TMT	Tandem mass tag
UTP15	U3 small nucleolar ribonucleoprotein15
