# Supplementary figures and images for: TMT-Based Quantitative Proteomic Analysis Reveals the Crucial Biological Pathways Involved in Self-Incompatibility Responses in Camellia oleifera
Source: Int J Mol Sci. 2020 Mar 14;21(6):1987. doi: 10.3390/ijms21061987 (PMC7139391; doi:10.3390/ijms21061987)

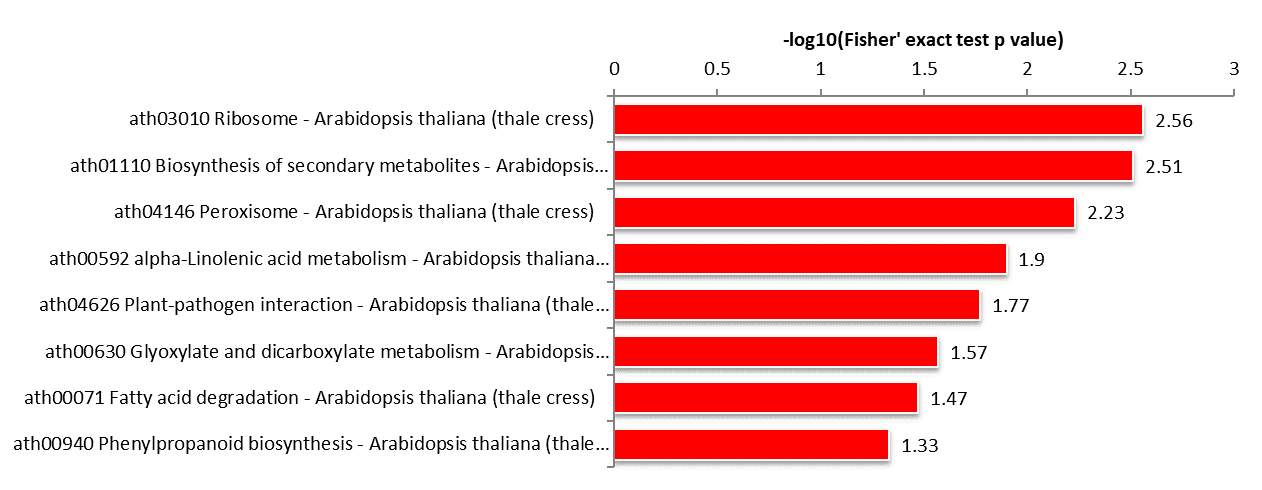

Supplement: Supplementary file 1 [file ijms-21-01987-s001.zip › supply material/Figure S10 KEGG enrichment analysis of DAPs between CP48 and CP65..png]

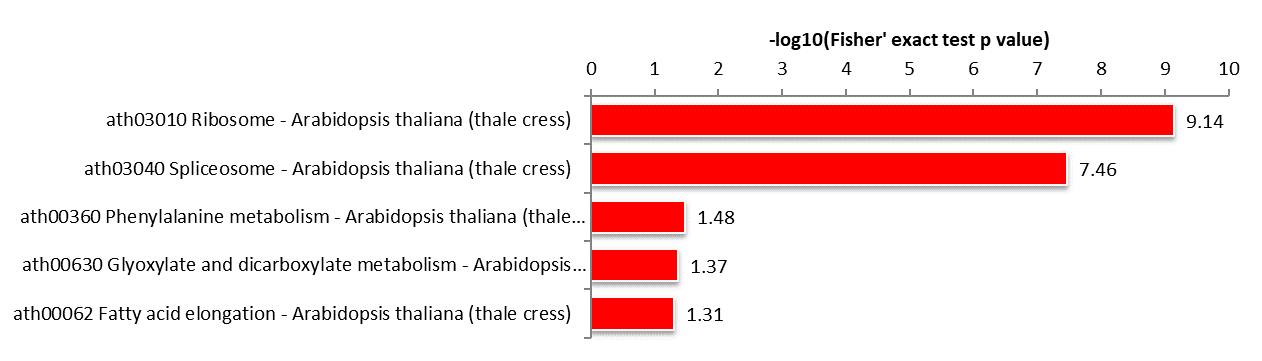

Supplement: Supplementary file 1 [file ijms-21-01987-s001.zip › supply material/Figure S11 KEGG enrichment analysis of DAPs between CP48 and CP75..png]

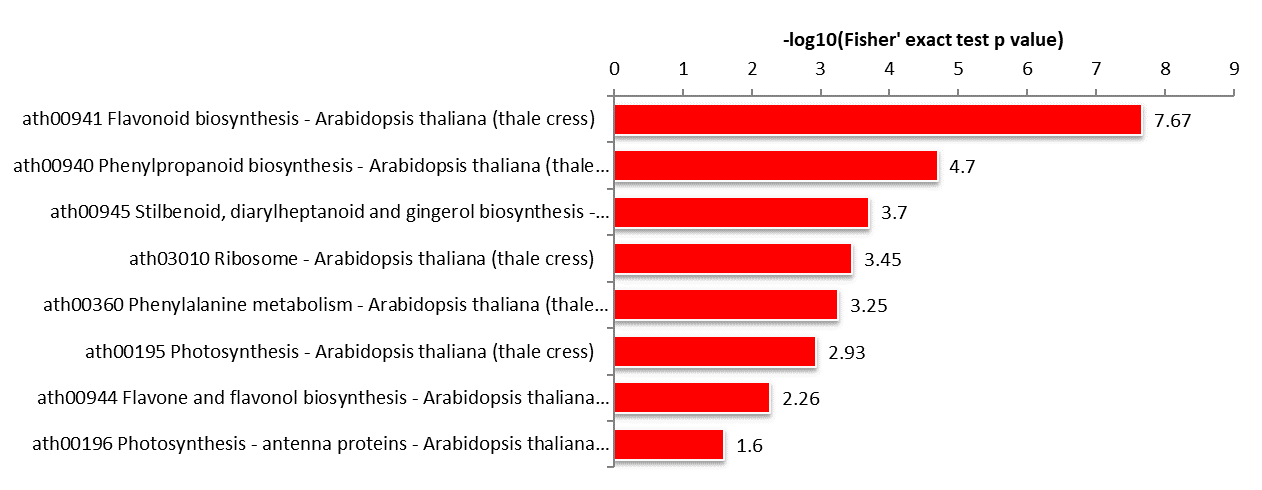

Supplement: Supplementary file 1 [file ijms-21-01987-s001.zip › supply material/Figure S12 KEGG enrichment analysis of DAPs between SP48 and SP65..png]

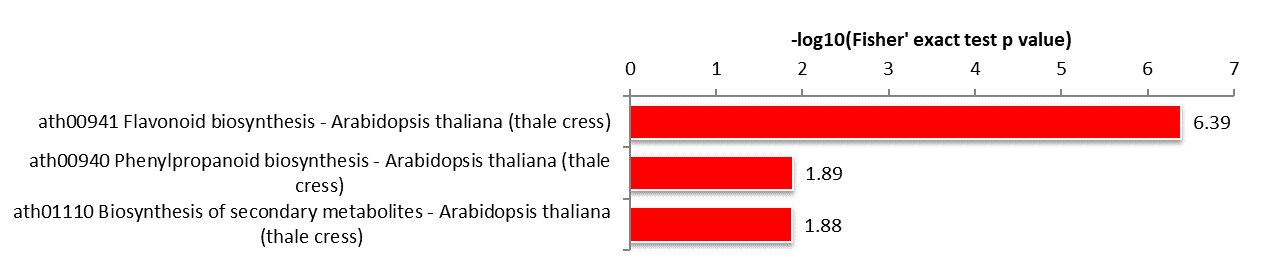

Supplement: Supplementary file 1 [file ijms-21-01987-s001.zip › supply material/Figure S13 KEGG enrichment analysis of DAPs between SP48 and SP75..png]

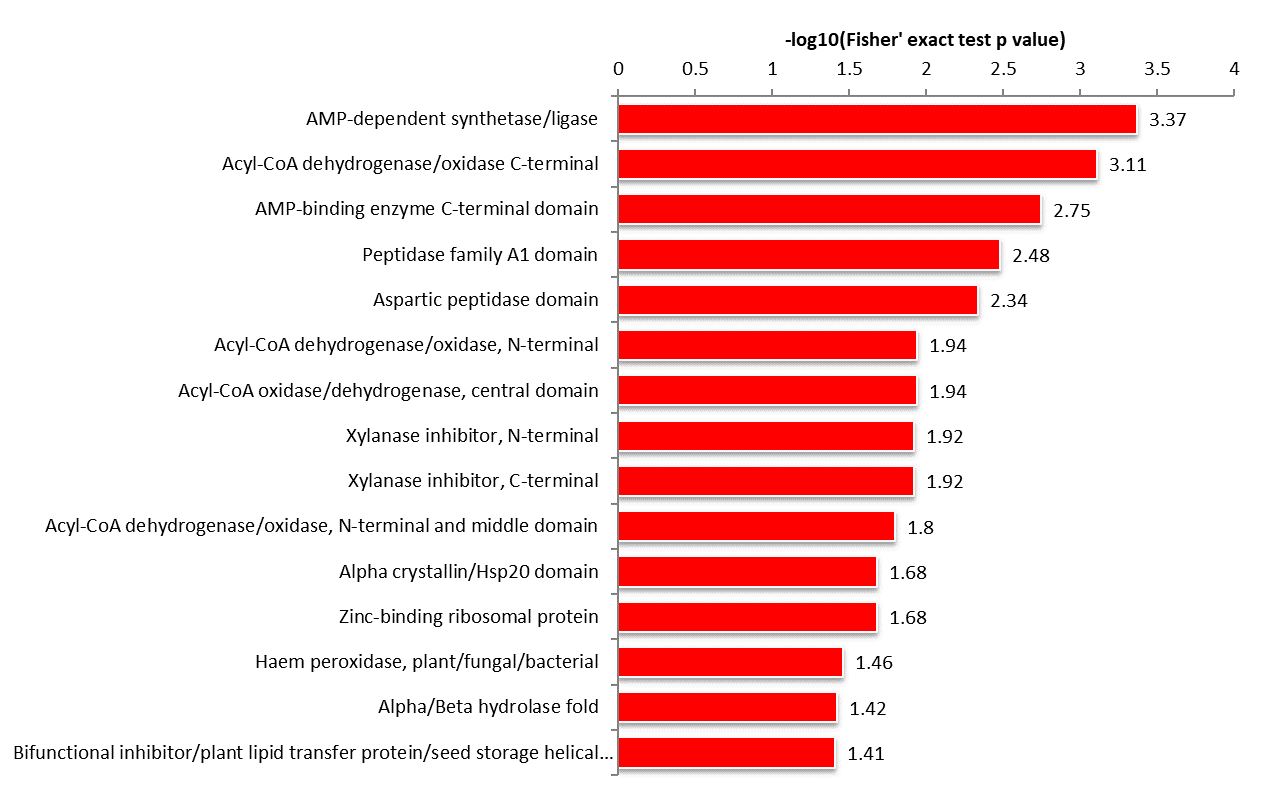

Supplement: Supplementary file 1 [file ijms-21-01987-s001.zip › supply material/Figure S14 Protein domain enrichment analysis of DAPs between CP48 and CP65..png]

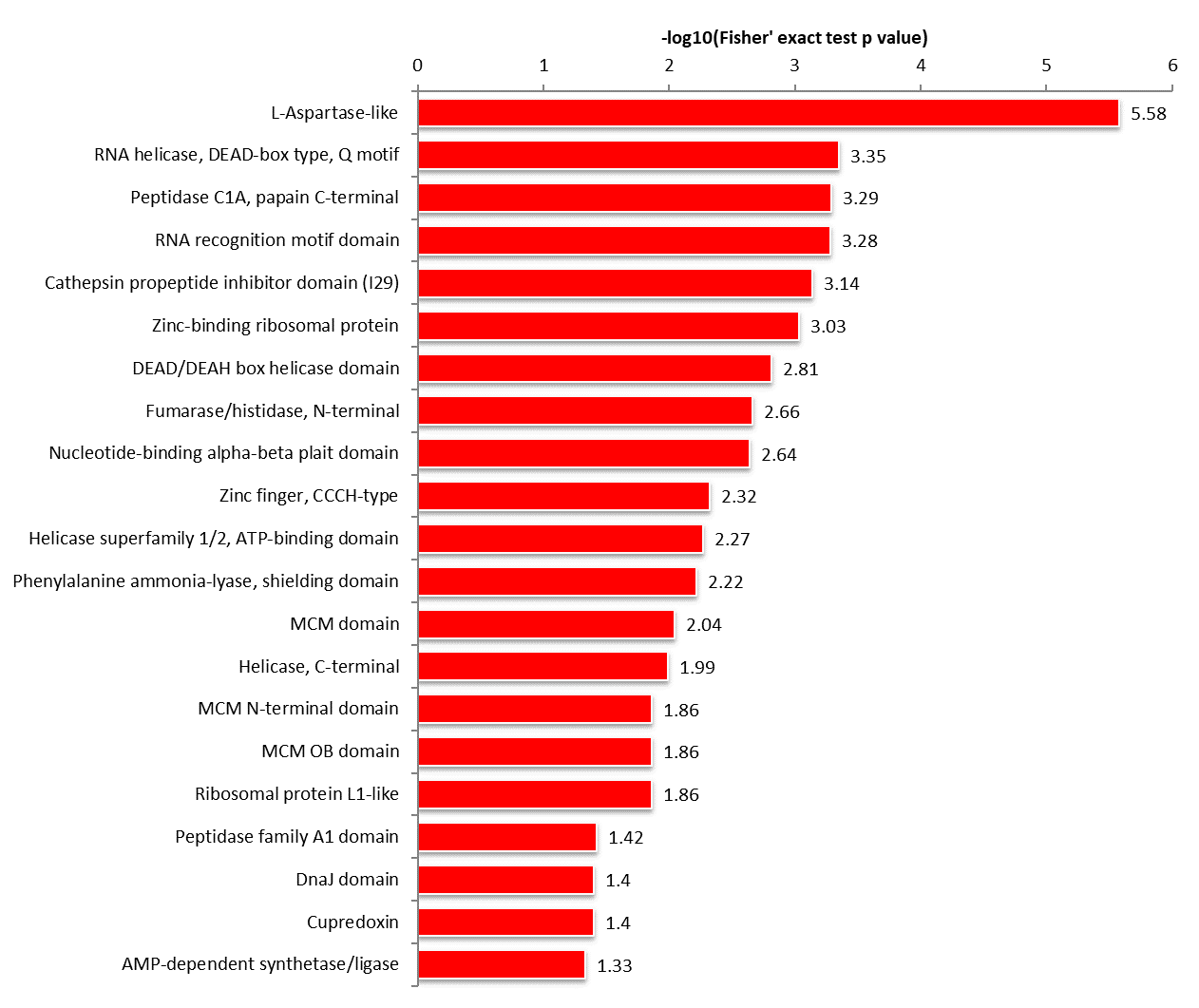

Supplement: Supplementary file 1 [file ijms-21-01987-s001.zip › supply material/Figure S15 Protein domain enrichment analysis of DAPs between CP48 and CP75..png]

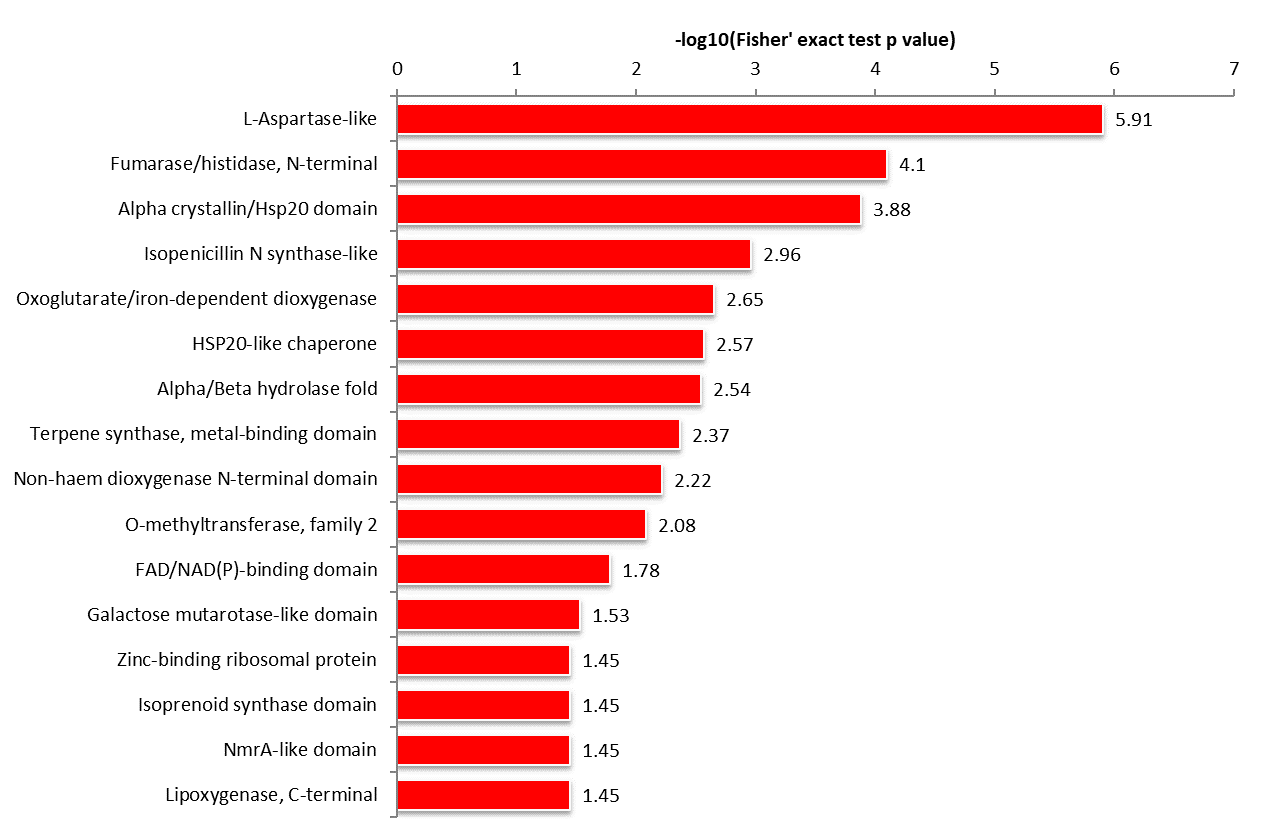

Supplement: Supplementary file 1 [file ijms-21-01987-s001.zip › supply material/Figure S16 Protein domain enrichment analysis of DAPs between SP48 and SP65..png]

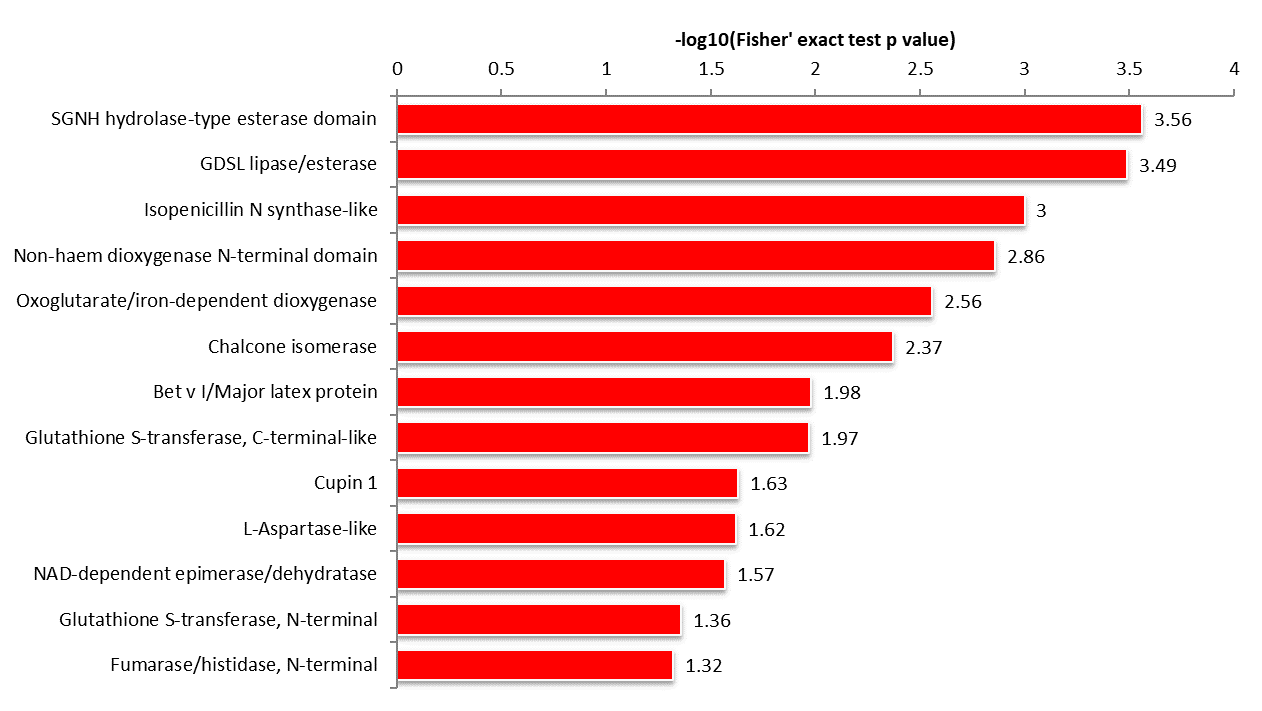

Supplement: Supplementary file 1 [file ijms-21-01987-s001.zip › supply material/Figure S17 Protein domain enrichment analysis of DAPs between SP48 and SP75..png]

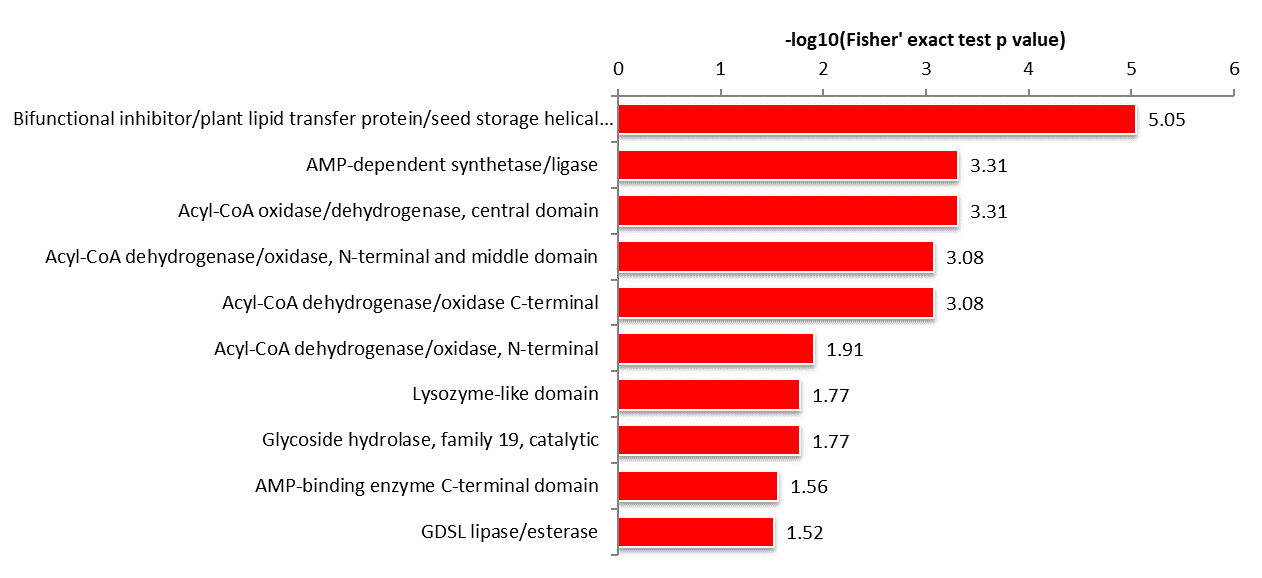

Supplement: Supplementary file 1 [file ijms-21-01987-s001.zip › supply material/Figure S18 Protein domain enrichment analysis of DAPs between CP48 and SP48..png]

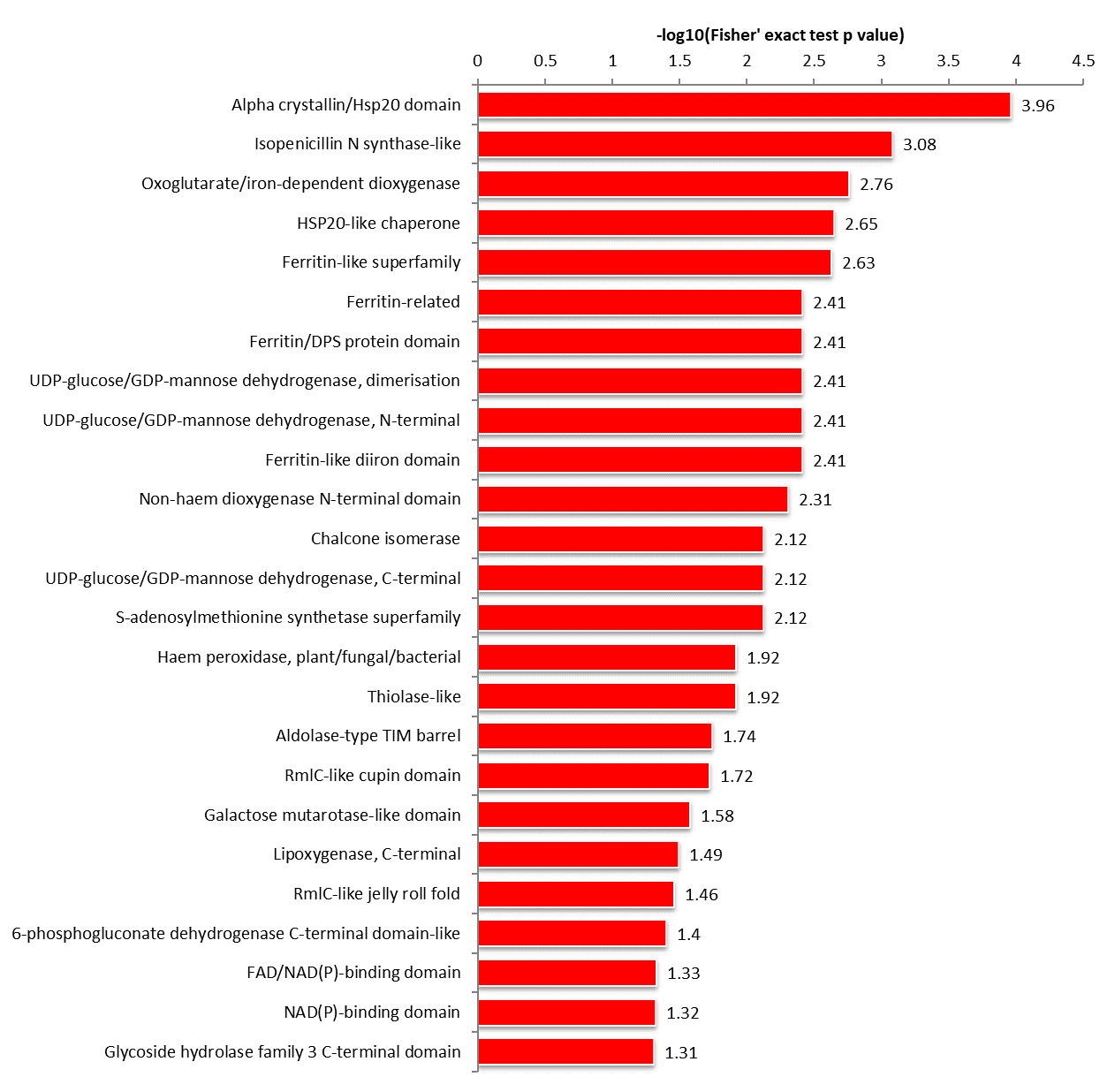

Supplement: Supplementary file 1 [file ijms-21-01987-s001.zip › supply material/Figure S19 Protein domain enrichment analysis of DAPs between CP65 and SP65..png]

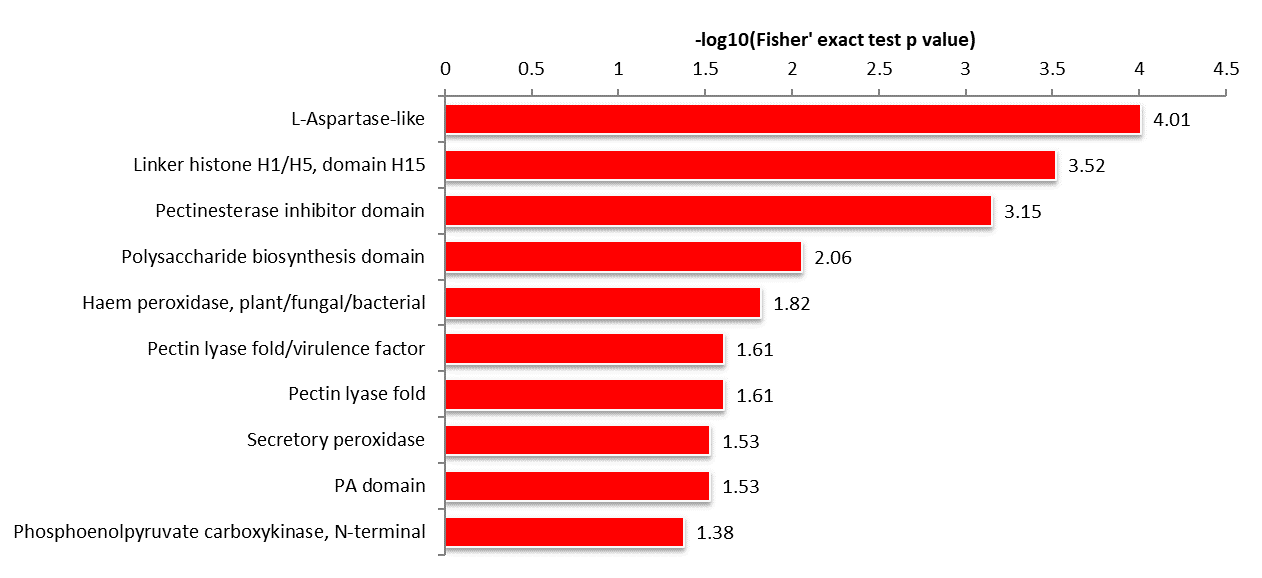

Supplement: Supplementary file 1 [file ijms-21-01987-s001.zip › supply material/Figure S20 Protein domain enrichment analysis of DAPs between CP75 and SP75..png]

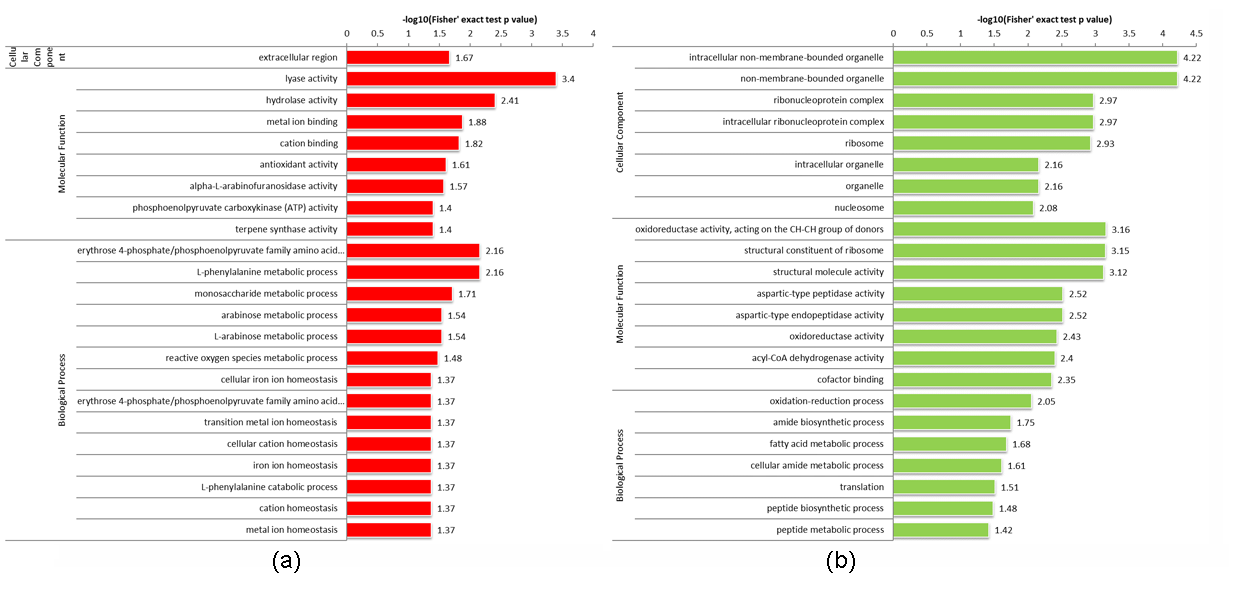

Supplement: Supplementary file 1 [file ijms-21-01987-s001.zip › supply material/Figure S3 GO enrichment analysis of DAPs between CP48 and CP65..tif]

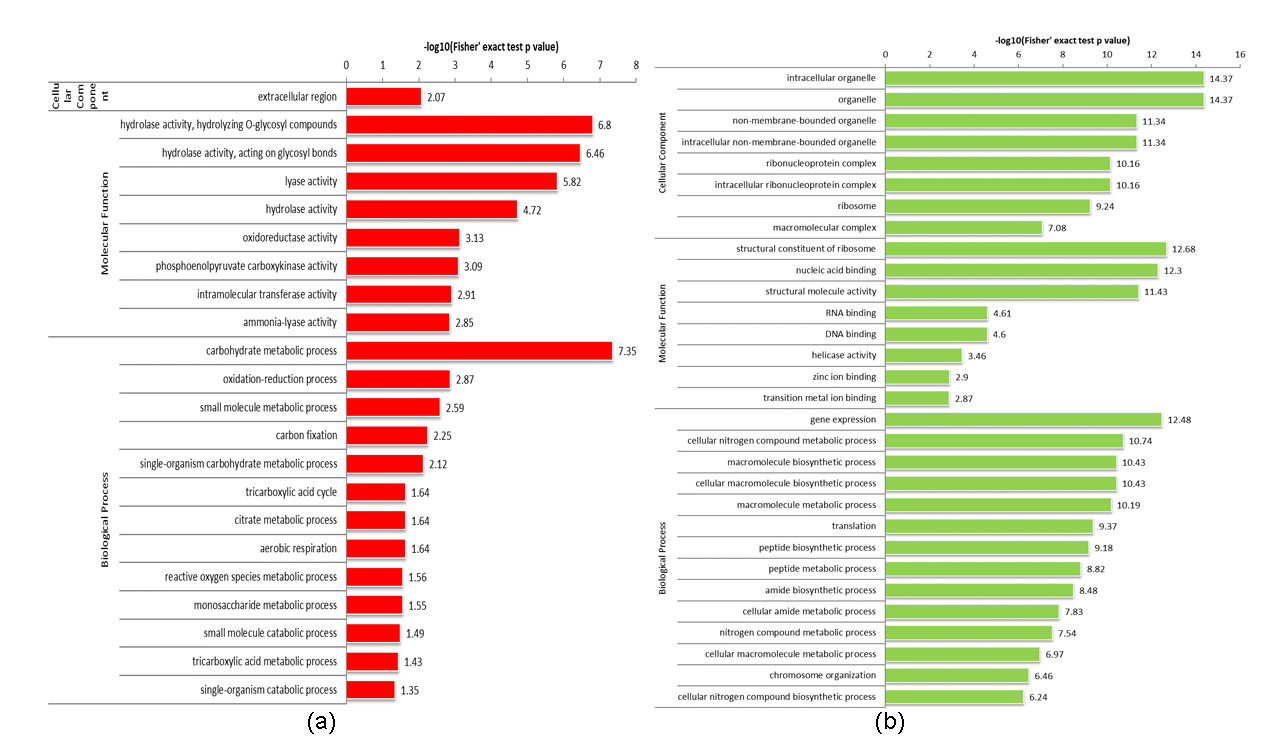

Supplement: Supplementary file 1 [file ijms-21-01987-s001.zip › supply material/Figure S4 GO enrichment analysis of DAPs between CP48 and CP75..tif]

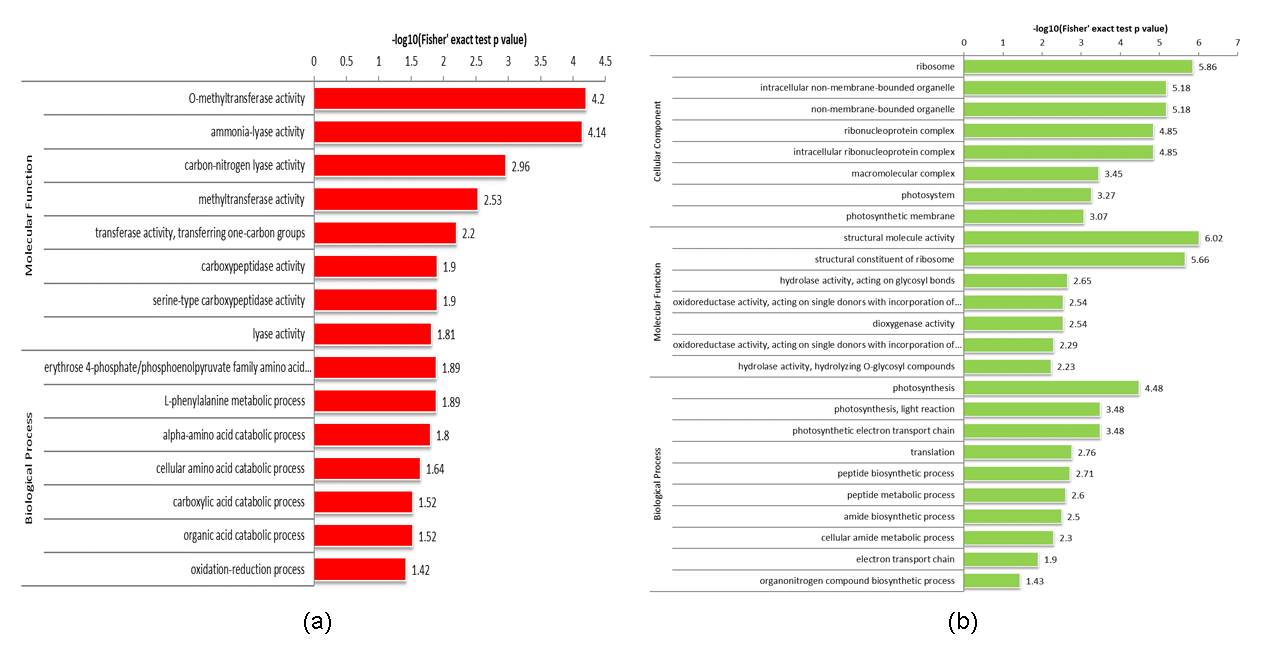

Supplement: Supplementary file 1 [file ijms-21-01987-s001.zip › supply material/Figure S5 GO enrichment analysis of DAPs between SP48 and SP65..tif]

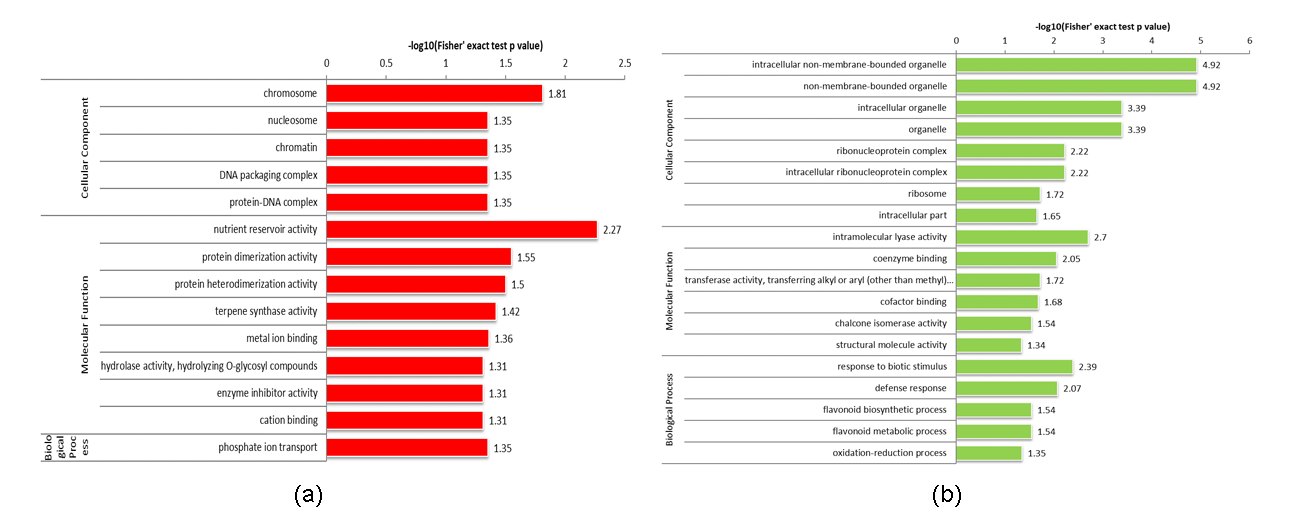

Supplement: Supplementary file 1 [file ijms-21-01987-s001.zip › supply material/Figure S6 GO enrichment analysis of DAPs between SP48 and SP75..tif]

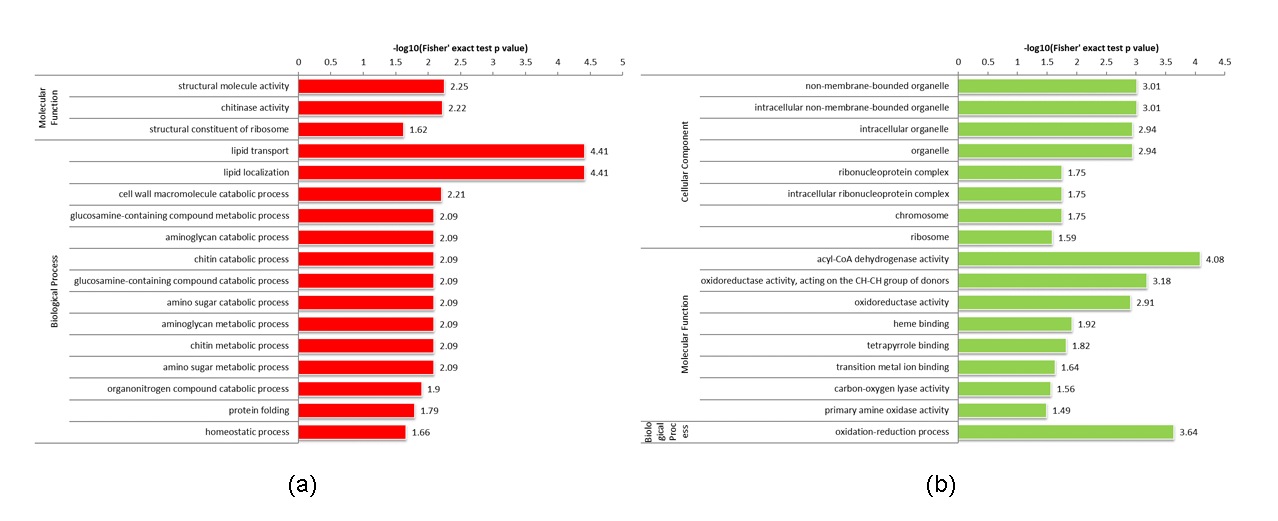

Supplement: Supplementary file 1 [file ijms-21-01987-s001.zip › supply material/Figure S7 GO enrichment analysis of DAPs between CP48 and SP48..tif]

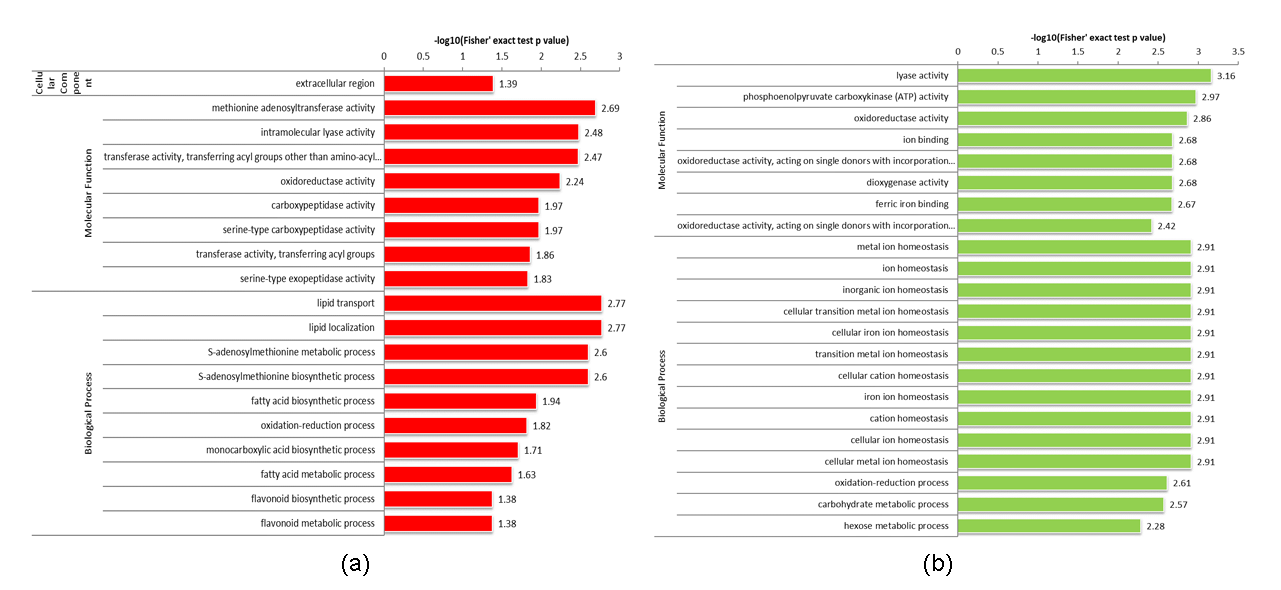

Supplement: Supplementary file 1 [file ijms-21-01987-s001.zip › supply material/Figure S8 GO enrichment analysis of DAPs between CP65 and SP65..tif]

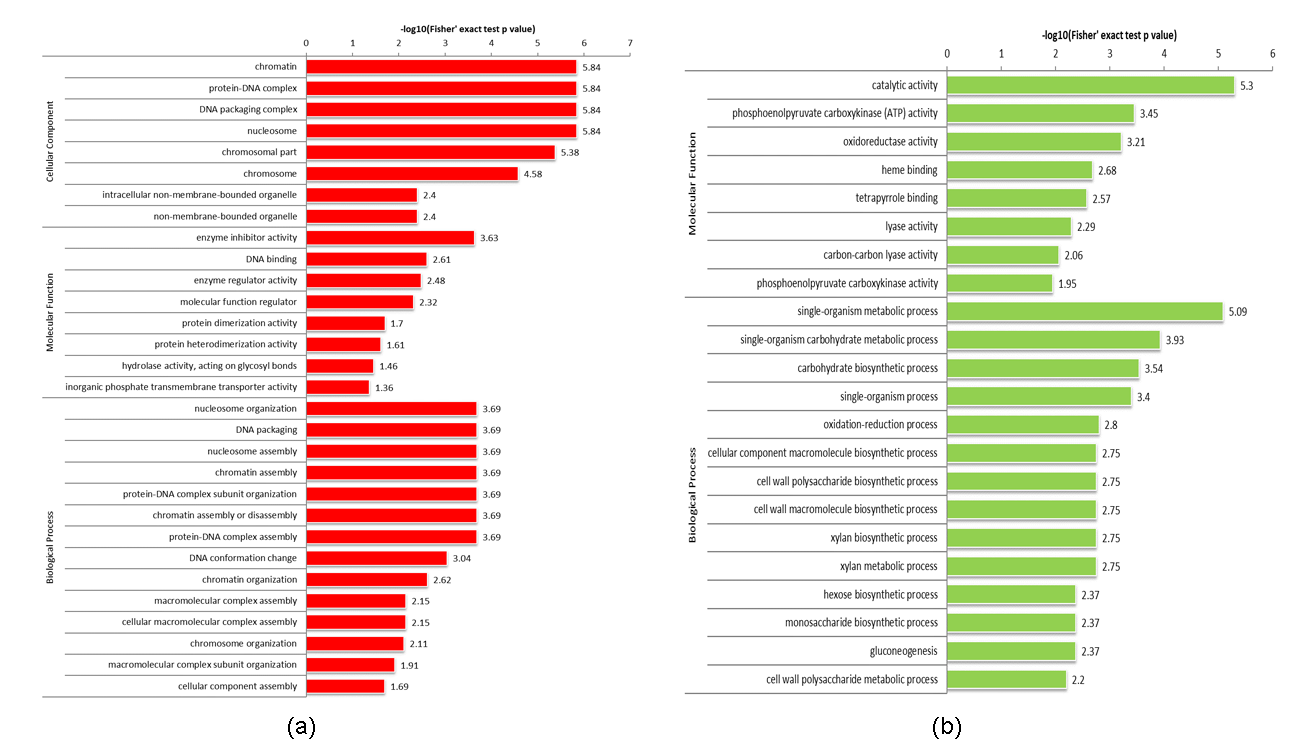

Supplement: Supplementary file 1 [file ijms-21-01987-s001.zip › supply material/Figure S9 GO enrichment analysis of DAPs between CP75 and SP75..tif]
